# A robust lateral shift free (LSF) electrothermal micromirror with flexible multimorph beams

**DOI:** 10.1038/s41378-023-00570-8

**Published:** 2023-08-29

**Authors:** Hengzhang Yang, Anrun Ren, Yingtao Ding, Lei Xiao, Teng Pan, Yangyang Yan, Wenlong Jiao, Huikai Xie

**Affiliations:** 1 School of Integrated Circuits and Electronics, Beijing Institute of Technology, Beijing, 100081 China; 2 BIT Chongqing Institute of Microelectronics and Microsystems, Chongqing, 400030 China

**Keywords:** NEMS, Sensors

## Abstract

Electrothermal bimorph-based scanning micromirrors typically employ standard silicon dioxide (SiO_2_) as the electrothermal isolation material. However, due to the brittle nature of SiO_2_, such micromirrors may be incapable to survive even slight collisions, which greatly limits their application range. To improve the robustness of electrothermal micromirrors, a polymer material is incorporated and partially replaces SiO_2_ as the electrothermal isolation and anchor material. In particular, photosensitive polyimide (PSPI) is used, which also simplifies the fabrication process. Here, PSPI-based electrothermal micromirrors have been designed, fabricated, and tested. The PSPI-type micromirrors achieved an optical scan angle of ±19.6° and a vertical displacement of 370 μm at only 4 Vdc. With a mirror aperture size of 1 mm × 1 mm, the PSPI-type micromirrors survived over 200 g accelerations from either vertical or lateral directions in impact experiments. In the drop test, the PSPI-type micromirrors survived falls to a hard floor from heights up to 21 cm. In the standard frequency sweeping vibration test, the PSPI-type micromirrors survived 21 g and 29 g acceleration in the vertical and lateral vibrations, respectively. In all these tests, the PSPI-type micromirrors demonstrated at least 4 times better robustness than SiO_2_-type micromirrors fabricated in the same batch.

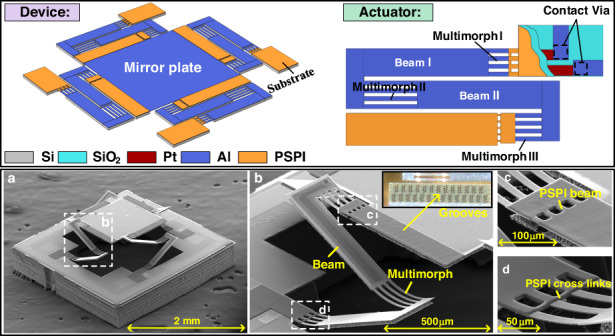

## Introduction

Microelectromechanical system (MEMS) scanning micromirrors have recently attracted broad interest due to their potential applications in LiDAR^1,2^, 3D cameras^3–5^, and VR/AR^6–8^. Several types of MEMS micromirrors exist, including electromagnetic, electrostatic, and electrothermal micromirrors^9,10^. Compared with electromagnetic and electrostatic micromirrors, electrothermal micromirrors use thermal bimorph actuators and offer the advantages of large displacement, large nonresonant angular scan range, high fill factor, and low driving voltage^11,12^. Thus, they have been applied in optical endoscopic imaging^13^ and Fourier transform near-infrared spectrometers^14^. The most commonly used bimorph actuator materials for electrothermal micromirrors are Al and SiO_2_ due to their wide availability and large differences in their coefficients of thermal expansion (CTE). SiO_2_, due to low thermal conductivity, is also used to provide thermal isolation at anchors to reduce power consumption^15^. However, electrothermal micromirrors with SiO_2_ anchors are susceptible to impact failure due to the brittle nature of SiO_2_. The typical fracture strength of thin-film SiO_2_ is only 0.77 MPa m^1/2^ (ref. ^16^), which is 1/40 that of aluminum^17^. As a result, such electrothermal micromirrors are easily damaged by accidental collisions or drops. For instance, some electrothermal micromirrors may not even survive a drop from a height of a few centimeters^18^. Experimental analyses have shown that device failure is mainly caused by the fracture of SiO_2_ anchors at the ends of bimorph actuators^19^. This susceptibility to impact failure largely limits the application range of electrothermal micromirrors, especially in hand-held optical imaging probes, which often experience accidental drops or collisions.

Some methods have been proposed to improve the impact resistance of electrothermal micromirrors. For instance, Pal et al. reported a simple bimorph-based one-dimensional (1D) electrothermal micromirror that used polyimide (PI) as a stress buffer^18^, but the unidirectional operation, nonstationary center of rotation, and large initial tilt angle of this 1D micromirror made the device packaging and optical design more complex and difficult^20^. Furthermore, the PI etching required a hard mask material, and the fabrication process was not applicable to make electrothermal micromirrors with more sophisticated bimorph actuators, such as triple-bimorph lateral-shift-free (LSF) actuators. Zhang et al. proposed an LSF 2D micromirror that used Cu/W bimorphs to replace Al/SiO_2_ bimorphs to improve its robustness^21^. However, it proved challenging to passivate Cu layers due to how Cu/W bimorphs must operate under high temperatures. In addition, fragile SiO_2_ was used for thermal isolation at anchor regions, such that the breakage issue was not obviated.

Here, a new fabrication process is presented for robust Al/SiO_2_ bimorph LSF 2D electrothermal micromirrors with flexible multimorph beams enabled by the incorporation of photosensitive polyimide (PSPI). In this new process, PSPI partially replaces SiO_2_ as the electrothermal isolation material and as the anchor material, making this new type of electrothermal micromirrors much more robust to impact and more power efficient. These new features can extend the applications of electrothermal mirrors to a much wider range, including portable or hand-held devices. In the following sections, the design, fabrication, and characterization of the micromirror are described.

## Results

### Design of the robust Al/SiO_2_-based LSF 2D micromirror

**Fig. 1:**
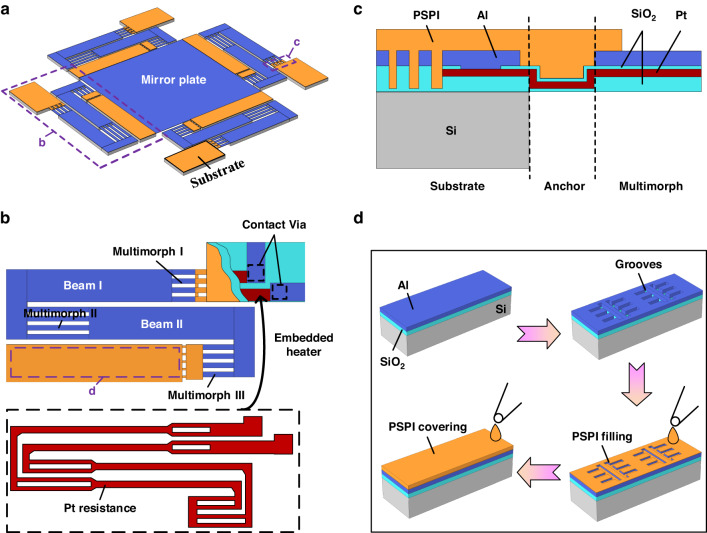
Schematic illustration of the LSF robust micromirror design. **a** Schematic 3D diagram of the LSF micromirror, which consists of a mirror plate, four actuators, and a substrate. **b** A top view of the LSF actuator, which consists of three multimorphs and two straight beams. Both ends of the actuator have PSPI anchors. **c** A cross-sectional view of the PSPI anchors connecting the actuators to the substrate. **d** A series of grooves are designed on the frame and substrate and filled with PSPI for better adhesion

A schematic 3D diagram of the robust tip-tilt-piston electrothermal micromirror is shown in Fig. 1a. The mirror plate is suspended by Al/SiO_2_-based LSF actuators (Fig. 1b) on the four sides. Each actuator has three multimorphs beams and two straight beams. The lengths of the three multimorphs beams are chosen such that the curlings of the first and third multimorphs beams are compensated by that of the second one. The two straight beams are fortified by the single-crystal silicon layer underneath, which is used to amplify the vertical displacement. A serpentine Pt resistance wire is embedded between the Al and SiO_2_ layers as a Joule heater. Both ends of the actuators are equipped with PSPI anchors, where one end is connected to the mirror plate and the other end is connected to the substrate. The cross-sectional view of the PSPI anchor on the substrate side is shown in Fig. 1c. There is no Si attached below the anchor, which significantly reduces the diffusion of the Joule heat generated on the multimorphs into the substrate and thus minimizes power consumption. Most importantly, the flexible PSPI anchors cushion external impacts by deforming, which may reduce the possibility of anchor fracture and device failure in response to such impacts.

To increase the adhesion between PSPI and the microstructures, PSPI must be coated on sufficiently large areas with grooves. A schematic of the PSPI coating process is shown in Fig. 1d. Grooves are preformed on the Al and SiO_2_ layers on a silicon wafer. Liquid PSPI is first spun on the grooves and other areas on the wafer, and then the wafer is held in a vacuum environment where the PSPI fills in the grooves. After, a second liquid PSPI spin coating is applied. Through UV exposure, the PSPI is completely cured in a temperature-controlled oven. Hereafter, a tenon-like PSPI structure is firmly adhered to the wafer and does not peel off.

In this new micromirror design, the PSPI connects the bimorph actuators to both the mirror plate and substrate as a mechanical connector, a stress absorber, a thermal isolator, and an electrical insulator. The mechanical, electrothermal properties of bimorph actuators are largely determined by the structural parameters of the PSPI. Mechanically, the main concerns are adhesion, stiffness, and stress relief. The adhesion is enhanced by designing multiple grooves in which the PSPI fills. The PSPI portion connecting the bimorph actuators to the substrate affects the overall stiffness of the bimorph actuation beams. The thickness of the PSPI film is determined by the polymer properties and the employed coating process. For our device, extensive experiments show the optimal thickness to be 4 µm. Regarding the width, it is important to keep the PSPI with the same width as that of the bimorph beams, maintaining the uniformity of the release etching process and the integrity of the bimorph actuation beams. Therefore, the choice of the PSPI length has some flexibility. Two aspects are deserving of consideration: stiffness and stress. Primarily, the incorporation of the PSPI must not substantially reduce the stiffness of the bimorph actuation beams. A simulation is carried out to evaluate the resonance frequency of the micromirror versus the PSPI length, as shown in Table S2. The length is chosen as 15 μm. With this design, the resonant frequency of the micromirror only decreased by 5% compared to its pure SiO_2_/Al counterpart. Moreover, the simulation shows that the 15-μm-long PSPI can effectively absorb the stress under 4 V actuation. Finally, since PSPI is a good electrothermal isolator with a maximum operating voltage of only 4 V, there are no concerns regarding the electrothermal properties.

In summary, there are several advantages offered by the electrothermal micromirror design shown in Fig. 1. First, this new design preserves all features of previous LSF micromirrors, including tip-tilt-piston scanning, a large scan range, a high fill factor, and a low driving voltage. Second, it overcomes the low impact resistance that previous LSF micromirrors suffered from by incorporating flexible polymer materials. Third, it increases the power efficiency, as the employed polymer has lower thermal conductivity than SiO_2_. Fourth, PSPI simplifies the fabrication process. Last, grooves have been designed to allow PSPI to fill in and thus to increase the adhesion between PSPI and the microstructures.

### Device fabrication process

**Fig. 2:**
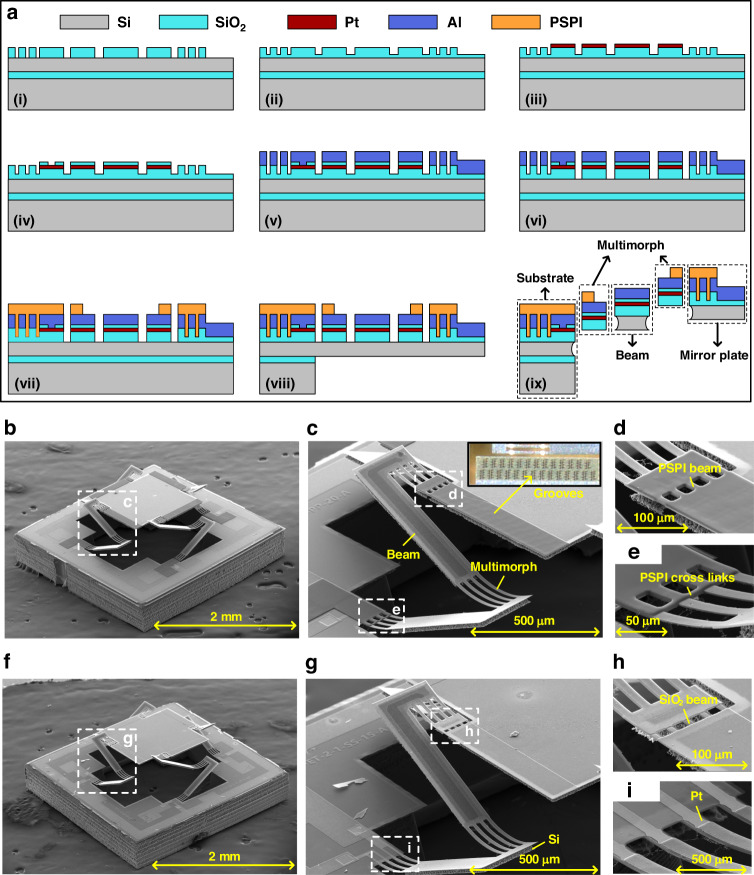
Fabrication process of the robust LSF electrothermal micromirror with flexible multimorph beams and released devices. **a** The fabrication process of the PSPI-type micromirror. **b** SEM images of the PSPI-type micromirror after release, where the mirror plate is lifted up by the bimorph beams via the residual stresses. Both ends of the actuators have PSPI structures as connections to the mirror plate and substrate. **c** Close view of the actuator. **d**, **e** Close views of the PSPI structures at the ends of the actuators. **f** SEM image of the SiO_2_-type micromirror. **g** Close view of an LSF actuator. **h**, **i** Close views of the SiO_2_ structures at the ends of the actuators

The micromirrors are fabricated on an SOI wafer using a combined surface and bulk micromachining process. The fabrication process is illustrated in Fig. 2a, which shows the cross-sectional view of a micromirror device. The detailed process is described as follows. In Fig. 2a(i), a 1 μm SiO_2_ layer is deposited on the SOI wafer with a plasma-enhanced chemical vapor deposition (PECVD) process at a temperature of 300°C, followed by a wet etching process to form part of the multimorph patterns. In Fig. 2a(ii), a 0.1 μm SiO_2_ layer is deposited. In Fig. 2a(iii), a 0.15 μm Pt layer is deposited using sputtering and patterned with a lift-off process. In Fig. 2a(iv), a 0.1 μm SiO_2_ layer is deposited to form electrical isolation on the multimorphs, followed by a reactive ion etching (RIE) process to form vias. In Fig. 2a(v), a 1 μm Al layer is deposited and dry etched to form the multimorph structures, the mirror plate, and the electrical wires distributed on the substrate. In Fig. 2a(vi), a 0.2 μm SiO_2_ layer is removed by RIE to define the shape of the multimorph actuators and dicing grooves.

The fabrication process of the flexible connect anchor is shown in Fig. 2a(vii), where PSPI (JAPB-101, Jingai Microelectronics Co., Ltd., China) is applied. The wafer is placed in a vacuum oven after coating to allow the PSPI to completely fill the grooves, as shown in Fig. 1d. These grooves provide the PSPI layer with a better attachment to the actuators. To ensure the uniformity of the PSPI on the wafer, a two-speed spin coating method is adopted, with a low speed of 1000 rpm for 20 s and a high speed of 3000 rpm for 40 s. Then, a 120 °C prebake on a hot plate for 5 min is carried out, followed by UV exposure. After that, the developer solution, which contained 2.38 wt% tetramethylammonium hydroxide (TMAH), was used for development at room temperature for 90 s, and then the wafer was placed in low-power oxygen plasma to remove the residual PSPI. Finally, the PSPI is completely cured in a nitrogen environment by a step heating progress from room temperature to 320 °C and is held at 320 °C for 120 min^22^, and then the wafer is gradually cooled down to room temperature.

After coating and patterning PSPI, 400 μm deep reactive ion etching (DRIE) of silicon on the handle layer and 1 μm buried oxide removal by RIE are carried out sequentially to facilitate device release (Fig. 2a(viii)). Finally, XeF_2_ is used to remove the residual silicon underneath the multimorphs and PSPI beams (Fig. 2a(ix)). Because XeF_2_ has extremely high etching selectivity to SiO_2_, Al, and PI^23–26^, the device structures exposed to the XeF_2_ environment are not damaged.

Figure 2b shows the PSPI-type electrothermal micromirror after release. The size of the mirror plate is 1 mm × 1 mm. The device footprint is 2.7 mm × 2.7 mm. The dimensional parameters of the PSPI have a crucial impact on the electrical, mechanical, and thermal properties of bimorph actuators and the robustness of the device. After carefully balancing these key factors, the width, length, and thickness of the PSPI were chosen as 20 μm, 15 μm, and 4 μm, respectively. The detailed thickness of PSPI is presented in Figure S2. More information about the dimensions of this MEMS micromirror is listed in Table 1. The mirror plate is elevated by 470 μm above the substrate surface after release due to the residual stress-induced curvature of the multimorphs. There is a 25-μm-thick Si layer under the mirror plate and the straight beams of the actuators. This Si layer ensures the optical flatness of the mirror plate and keeps the straight beams rigid, as shown in Fig. 2c. Figure 2d, e shows a close view of the PSPI thermal isolation structures at both ends of the actuators. Furthermore, a series of PSPI cross-links between the multimorph beams, as shown in Fig. 2d, may help the PSPI to be firmly attached to the multimorph structures.

**Table 1:** Designed parameters of the micromirrors

Structure parameter	Value
Device footprint	2.7 mm × 2.7 mm
Mirror plate size	1 mm × 1 mm
Length of Multimorph I/III	120 μm
Length of Multimorph II	240 μm
Length of Beam	560 μm
Length of PSPI	15 μm
Width of Multimorph	20 μm
Width of Pt resistance	10 μm
Width of PSPI	20 μm
Width of Beam	125 μm
Thickness of SiO_2_	1 μm
Thickness of Al	1 μm
Thickness of Pt	0.15 μm

We also fabricate electrothermal micromirrors with SiO_2_ as the connection anchor material. These are referred to as SiO_2_-type micromirrors. Except for the connection anchor structure, the SiO_2_-type micromirrors have exactly the same structural dimensions as the PSPI-type MEMS micromirrors. SEM images of the SiO_2_-type micromirror are shown in Fig. 2f–i.

When the same driving signal is applied on all four actuators, the micromirror works in the piston mode. One pair of opposing actuators can be activated to perform one-axis rotational scanning, while the other pair can generate orthogonal axis rotational scanning. Thus, the micromirror can work in the piston scan or two-axis angular scan mode.

### Static and dynamic response

**Fig. 3:**
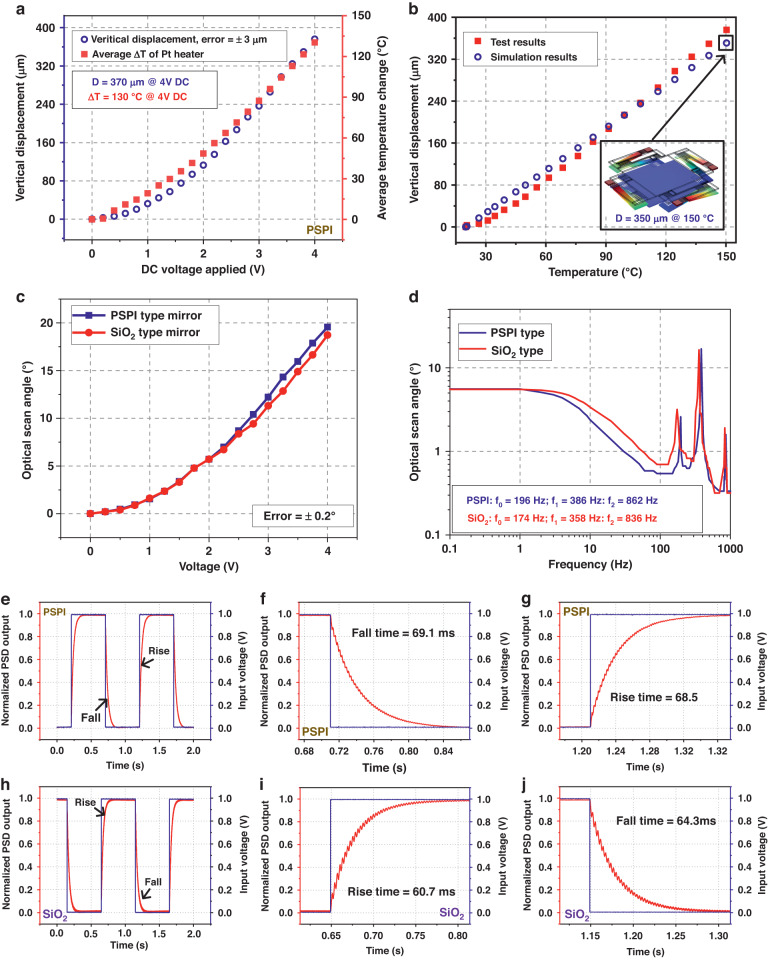
The static and dynamic responses of the two kinds of micromirrors. **a** Vertical displacement and average temperature change versus applied DC voltage of the PSPI-type micromirror. D: The displacement of the mirror plate. ${\Delta{\rm{T}}}$: the average temperature change value. **b** Test and simulation results of the vertical displacement versus the average temperature value for the PSPI-type micromirror. The room temperature was 20 °C. **c** Optical scan angle versus applied DC voltage by actuating an actuator. **d** Frequency response with a sinusoidal voltage at 0–2 V from 0.1 to 1000 Hz. **e** Step response of the PSPI-type micromirror. **f** Magnified picture of the fall time (cooling down). **g** Magnified picture of the rise time (heating up). **h** Step response of the SiO_2_-type micromirror. **i** Magnified picture of the rise time (heating up). **j** Magnified picture of the fall time (cooling down)

Joule heating is generated by the Pt resistors. The measured resistance of the heaters in the four actuators of the PSPI-type micromirror is 520 ± 20 Ω. The temperature coefficient of resistivity (TCR) of Pt is measured using a temperature-controlled oven, and the measured value of the TCR is 0.0022/K (Figure S1). The measured vertical displacement of the mirror plate versus the driving voltage is plotted in Fig. 3a, where the displacement reaches 370 μm at only 4 V DC, and good linearity is observed for the displacement range from 90 to 370 μm. Since the resistance of the embedded Pt heater increases significantly with temperature, the average temperature of an LSF actuator can be inferred by tracking the change in the Pt resistance. Figure 3a also shows the temperature change versus the applied voltage. As shown in Fig. 3b, the vertical displacement of the mirror plate increases approximately linearly with temperature, and the test results show favorable agreement with the simulation results.

The optical scan angle is measured using a position-sensitive detector (PSD). When a laser beam points at a working micromirror, the PSD records the position of the laser spot reflected from the mirror plate, and then the scan angle can be deduced. Figure 3c shows the optical scan angle versus the DC voltage applied to one of the actuators, where the optical scan angles at 4 V DC are 19.6° for the PSPI-type and 18.8° for the SiO_2_-type. These angular scan ranges can satisfy many application requirements, including endoscopic OCT imaging. The applied voltage can exceed 4 V DC, but nonlinearity and plastic deformation may occur at this condition^11,19^. Likewise, 4 V DC meets the human body safety requirement. Thus, in this study, we limited the applied voltage up to 4 V DC.

To measure the frequency response, a sinusoidal voltage varying between 0 and 2 V is applied to one of the actuators of the micromirror. The test results are shown in Fig. 3d, where three resonant peaks are observed in the 0.1–1000 Hz frequency scan range. As even higher order modes show minimal responsivity, we limited our frequency response up to 1 kHz. For the PSPI-type micromirror, the three peaks are 196, 386, and 862 Hz. With a fundamental resonance frequency of 196 Hz, the micromirror provides sufficient bandwidth for many endoscopic imaging applications. With a Q factor of approximately 50, the 3 dB cutoff frequency is only 5 Hz, and the corresponding thermal time constant *τ* = 31.8 ms based on ${\rm{\tau }}=1/(2{\rm{\pi }}{f}\!_{{cutoff}})$. In this case, ${\rm{\tau }}\,\cdot\, {f}_{0}=6.3$, which is close to 8, so nearly no overshoots are observed in the step response^27^.

The step responses of the PSPI-type micromirrors are measured with a square wave applied to one of the actuators. These are shown in Fig. 3e–g, indicating that the rise time is 68.5 ms and the fall time is 69.1 ms. The fall time is 0.6 ms longer, which is attributed to the back-flow heating effect from the mirror plate^21^. In contrast, the response times of the SiO_2_-type micromirror are slightly smaller than those of the PSPI-type micromirror, as shown in Fig. 3h–j. Note that the fall time of the PSPI-type micromirror is longer than that of the SiO_2_-type micromirror because PSPI has a smaller thermal conductivity than SiO_2_ (Table S1).

### Impact test with Newton’s cradle

**Fig. 4:**
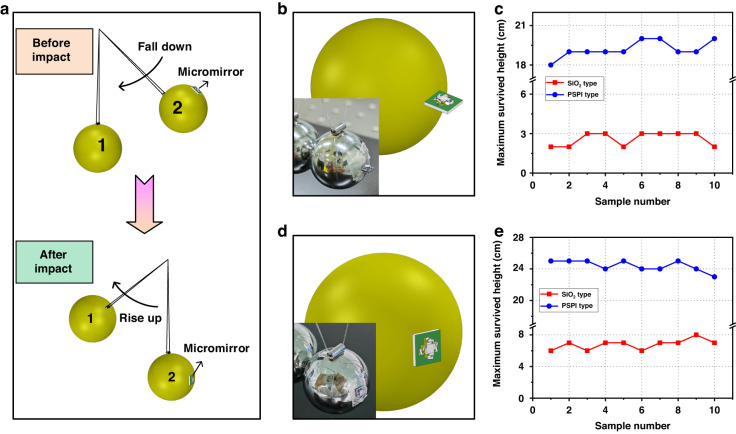
Impact test for SiO_2_ and PSPI type micromirrors with a Newton’s cradle. **a** Test setup (before and after the impact). **b** The micromirror is fixed on the ball for lateral impact from Newton’s ball. **c** The lateral impact test results of two types of micromirrors. **d** The micromirror is fixed on the ball for vertical impact from Newton’s ball. **e** The impact test results of two types of micromirrors

As illustrated in Fig. 4a, MEMS mirror testing occurs with fixation on one of the balls in an off-shelf Newton’s cradle. The micromirror can be placed to receive the impact from either the vertical or lateral direction. The masses of the micromirror device and one of the balls are measured using a high-precision balance, which are 0.46 mg and 44.0 g, respectively. The diameter of each ball is 2 cm. Upon testing, ball 2 is lifted and held at a height while ball 1 hangs down and stays stationary. Then, ball 2 is released and accelerates to hit the stationary ball 1. After the first impact, ball 1 is caught to prevent a successive collision. The lifted height determines the maximum acceleration. The height is increased by 1 cm after each test until the device is damaged. The health status of the micromirror after impact can be directly observed by eye. The maximum acceleration, ${a}_{{\rm{max }}}$, in m/s^2^ experienced by the device is given by^28^

1 $${a}_{{\rm{max }}}={v}_{{\rm{max }}}/\tau$$

2 $${v}_{{\rm{max }}}=\sqrt{2{gh}}$$

3 $$\tau =3.29{(1-{\sigma }^{2})}^{\frac{2}{5}}{({M}^{2}/R{E}^{2}{v}_{{\rm{max }}})}^{\frac{1}{5}}$$

where *τ* is the collision time of two balls, *h* is the drop height (m) for ball 2, *g* is the acceleration due to gravity (m/s^2^), ${v}_{{\rm{max }}}$ is the maximum velocity for ball 2, *M* (g) and *R* (m) are the mass and radius of each ball, respectively, and *σ* and *E* are Poisson’s ratio and Young’s modulus of the material of the balls. These balls are made of stainless steel, from which the material properties are taken^29^. For lateral impact, the mirror plate is parallel to the radial direction of the ball, which is shown in Fig. 4b. Figure 4c displays the test results. Nearly all the SiO_2_-type mirrors are damaged when the drop height exceeds 2 cm, corresponding to a maximum survival acceleration of only 54 g. In contrast, the PSPI-type micromirror withstands impact from the ball at a drop height of more than 19 cm, corresponding to a maximum survival acceleration of 209 g. For vertical impact, the mirror plate is perpendicular to the radial direction of the ball, which is shown in Fig. 4d. The test results are shown in Fig. 4e. The average survival drop heights of the SiO_2_-type and the PSPI-type micromirrors are 6 cm and 24 cm, respectively, and the corresponding maximum survival accelerations are 104 g and 240 g, respectively.

In the experiments involving both impact directions, the PSPI-type micromirror shows vastly improved robustness compared to the SiO_2_-type micromirror. Additionally, the impact resistances of both micromirrors in the vertical direction are better than those in the lateral direction. This is because the mirror plate is free to move in the vertical direction while constrained in the lateral direction.

### Drop test

**Fig. 5:**
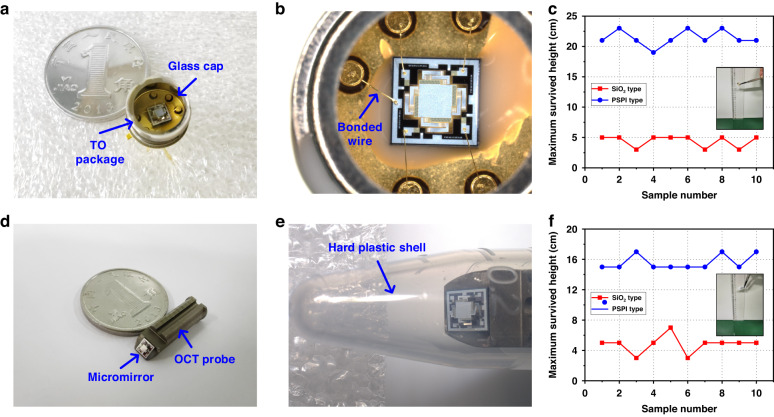
Drop test for the SiO2-type and PSPI-type micromirrors. **a** Photograph of the micromirror after encapsulation in the TO package. **b** Close view of the micromirror with bonded wires. **c** The drop test results of both types of micromirrors encapsulated with TO packages. **d** The micromirror is fixed on the OCT probe. **e** Photograph of the OCT probe after encapsulation with a transparent hard plastic shell. **f** The drop test results of two types of OCT probes encapsulated with a transparent hard plastic shell

The impact tests given by Newton’s cradle may only simulate a single impact event, whereas micromirrors in real-world use may suffer from many complex impact loads. Thus, drop tests presented in Fig. 5 are employed to further assess the robustness of the two types of micromirrors. All drop tests described in this section are performed on an Epoxy hard floor in a clean room. A micromirror is wire bonded in a transistor outline (TO) package, as shown in Fig. 5a, b, where a cover glass is used to eliminate the influence of the air flow on the mirror plate during the drop test. The total weight of the TO package is 0.89 g, which is measured by a high-precision balance. The resistances of the Pt heaters in the four actuators of each micromirror are measured before the drop test. The experiment begins with a drop height of 1 cm, from which the package falls randomly. Then, the test continues by increasing the height by 2 cm until the micromirror is damaged. After each drop, the micromirror is observed under an optical microscope. The micromirror is considered damaged if the mirror plate breaks off or if one or more actuator beams break. The test result is shown in Fig. 5c, where most of the SiO_2_-type micromirrors cannot even survive drops from a height of 5 cm. In contrast, nearly all of the PSPI-type micromirrors still work normally after free falling from a height of 21 cm. Detailed experimental data about the PSPI-type micromirror resistances and resonant frequencies after different drop heights are given in Table S3.

Electrothermal micromirrors can be widely used in OCT endoscopic imaging. OCT probes may inevitably suffer from accidental falling in actual operation, so the robustness of the micromirror is very important for the service life of OCT probes. An experiment was established to imitate the free-falling motion of the two types of micromirrors that are encapsulated in an OCT probe. A customized transparent and hard plastic shell is used for packaging to observe the micromirror after the OCT probe free falls. Figure 5d, e shows photos of the micromirror in the OCT probe before and after packaging (the size of the probe is 18 mm long and 5 mm in diameter). The total weight of the package is 2.2 g. The drop test is repeated for this structure using the previously mentioned method. The results are shown in Fig. 5f. For the SiO_2_-type micromirror, the device is damaged after free falling just when the height exceeds 5 cm. In contrast, the PSPI-type micromirror can withstand fall heights greater than 15 cm. Compared to the SiO_2_-type micromirror, the PSPI-type micromirror may offer greater potential in practical applications due to its improved robustness.

### Vibration test

**Fig. 6:**
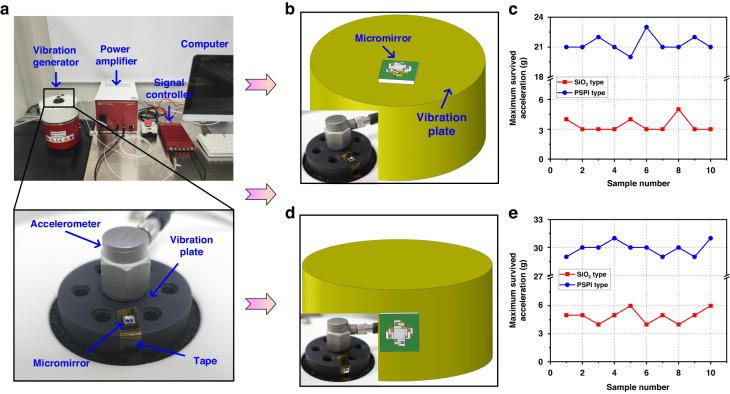
Vibration test for SiO_2_ and PSPI micromirrors. **a** The overall appearance of the vibration system and a close view of the vibrating plate. **b** The micromirror is fixed on the top plate for vertical vibration of the mirror plate. **c** The vertical vibration test results of two types of micromirrors. **d** The micromirror is fixed on the sidewall for vibration of the mirror plate. **e** The parallel vibration test results of two types of micromirrors

In practical applications, environmental vibrations also bring challenges to the normal operation and service life of the micromirror. A vibration test system (SignalCalc 901 DP), as shown in Fig. 6a, is employed here to test device robustness against this perturbation. Similar to Newton’s cradle test, in this vibration test, the influences of the vibrations from the vertical and parallel directions are studied on both types of micromirrors. The micromirrors are fixed on the vibration platform with double-sided adhesive tape, and the platform has only one degree of freedom in the Z direction. Figure 6b, d shows two fixing modes of the micromirror, corresponding to the vertical and horizontal vibrations, respectively. The vibration of the platform is excited with a sinusoidal signal with varying frequency and amplitude. Following the JESD22-B103B standard^30^, the vibration frequency is set to sweep from 20 to 2000 Hz and then back to 20 Hz, which is increased on a logarithmic scale, and the duration is set to 12 min. The acceleration amplitude is increased by 1 g after each frequency sweep until the device is damaged. During the whole process of vibration, the health status of the micromirror can be directly observed with the naked eye.

Figure 6c shows the results of the micromirrors vibrating in the vertical direction. Most of the SiO_2_-type micromirrors fail after the acceleration exceeds 3 g, while the PSPI-type micromirrors withstand more than 21 g. In addition, similar results are observed for horizontal vibrations, as are shown in Fig. 6e. Most of the SiO_2_-type micromirrors still cannot survive after the acceleration exceeds 5 g, while the PSPI-type micromirrors can withstand at least 29 g acceleration. Compared with the SiO_2_-type micromirrors, the PSPI-type micromirrors exhibit much improved vibration resistance in both vertical and horizontal directions, and their safe acceleration range is increased by at least 4–5 times. These experimental results show that the micromirrors are easier to damage when vibrating in the vertical direction, which is opposite from the cases of the impact test and drop test. More study is needed to fully understand this phenomenon.

## Discussion

**Fig. 7:**
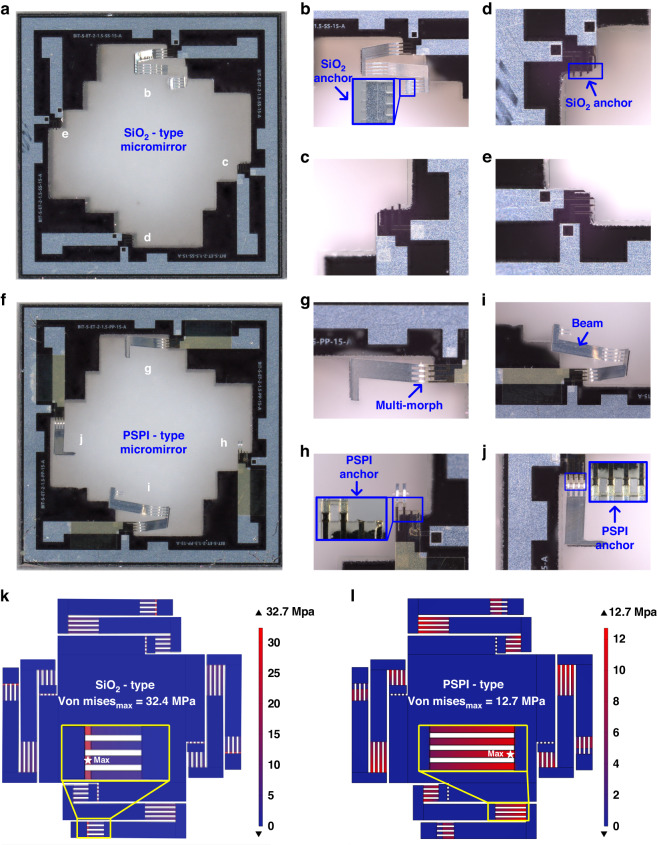
Comparison of the failure positions of two types of micromirrors and simulation analysis results. **a** Whole device of the damaged SiO_2_-type micromirror. **b**–**e** Close views of the failed positions of SiO_2_-type micromirrors. **f** Whole device of the damaged PSPI-type micromirror. **g**–**j** Close views of the failed positions of PSPI-type micromirrors. **k** Simulation results of the stress distribution of the SiO_2_-type micromirror. **l** Simulation results of the stress distribution of the PSPI-type micromirror

We introduced a new 2D LSF micromirror with flexible PSPI anchors in the previous sections. To assess the robustness of the PSPI-type micromirror, Newton’s cradle impact test, hardfloor drop test, and standard vibration test were conducted. All the experimental results show that the impact resistance of the new PSPI-type micromirrors is greatly improved compared to that of conventional SiO_2_-type micromirrors. Representative photos of the damaged SiO_2_-type and PSPI-type devices are shown in Fig. 7a–e and 7f–j, respectively. After carefully examining these photos, it is interesting to note that the fracture positions of the two types are different. For the SiO_2_-type micromirrors, the anchor structures are composed of only one layer of 1-μm-thick SiO_2_, such that they are easily damaged due to the brittle nature of SiO_2_. The breakage occurs either at the joint to the mirror plate (see Fig. 7b) or at the anchor to the substrate (see Fig. 7c–e). For the PSPI-type, the breakage positions appear at the middle points of the multimorph actuators (see Fig. 7g–j). In other words, the most easily damaged point on the actuator is changed from the anchor points in the SiO_2_-type to the connection points between the multimorphs and the straight beams in the PSPI-type.

3D models of both types of micromirrors have been built in the COMSOL software to determine the stress distribution difference between these two types. A 5 g acceleration load is applied in the z-direction in the simulation. Figure 7k, l show the simulation results of the SiO_2_ type and PSPI type, respectively. For the SiO_2_-type micromirror, the maximum stress point is unsurprisingly located at the thermal isolation anchor point, and the maximum Von Mises stress is 32.4 MPa. For the PSPI-type micromirror, the position of the maximum stress point is located at the junction of multimorph II and straight beam II, and the maximum Von Mises stress is 12.7 MPa. Note that at this junction location, the Von Mises stress in the SiO_2_-type micromirror is also 12.7 MPa. Structurally, the difference between these two types of micromirrors lies in the anchor regions where PSPI is adapted in the PSPI-type. It is clear that PSPI effectively reduces the stress, such that the PSPI-type device exhibits greatly reduced stress at the anchor regions. On the other hand, the junctions between the multimorphs and the straight beams in both types are still made of the same SiO_2_ film, so the stresses in the middle of the actuators in both types remain large. As a result, the PSPI film successfully prevents device breakage at the anchor points. However, when the acceleration continues to increase, the stress-concentration points in the middle of the actuators eventually fail. Therefore, to further improve the impact resistance, PSPI may be incorporated in the junctions in the middle of the actuators. More details about the damaged device are shown in Figure S3. Furthermore, we also discuss the temperature distribution on PSPI beams located at both ends of the actuators. The simulation results are shown in Figure S4.

## Conclusion

A robust tip-tilt-piston LSF electrothermal micromirror design with flexible multimorph beams is designed and experimentally verified. In this new micromirror design, a photosensitive polyimide (PSPI) is applied to replace SiO_2_ as a stress buffering anchor material. Due to only requiring a one-step patterning process without the need for a hard mask, the PSPI micromirror fabrication process is greatly simplified. A static vertical displacement up to 370 μm and a scan optical angle of ±19.6° were achieved at only a 4 V DC voltage. In the three robustness tests, the PSPI-type micromirrors exhibited much improved impact resistance compared to the SiO_2_-type micromirror of approximately 4–5 times better tolerance to acceleration forces. Very interestingly, incorporating PSPI in the anchor regions that are the weakest points exposes the second weakest points to failure modes, an observation that agrees with simulated results. Thus, our future work will focus on improving the impact resistance of those regions. Broadly speaking, PSPI-type micromirrors can be widely used in handheld or portable scanning devices.

### Supplementary information

Supplementary Information
