# Supplementary material for: A robust lateral shift free (LSF) electrothermal micromirror with flexible multimorph beams
Source: Microsyst Nanoeng. 2023 Aug 29;9:108. doi: 10.1038/s41378-023-00570-8 (PMC10465609; doi:10.1038/s41378-023-00570-8)
Supplement: Supplementary file 1 — Supplementary Information [file 41378_2023_570_MOESM1_ESM.docx]

**Supplementary information**

**A Robust Lateral Shift Free (LSF) Electrothermal Micromirror With Flexible Multimorph Beams**

Hengzhang Yang ^1^, Anrun Ren ^1^, Yingtao Ding ^1^, Lei Xiao ^1^, Teng Pan ^1^, Yangyang Yan ^2^, Wenlong Jiao^2^ and Huikai Xie ^1,2^ *

^1^School of Integrated Circuits and Electronics, Beijing Institute of Technology, Beijing 100081, China

^2^BIT Chongqing Institute of Microelectronics and Microsystems, Chongqing 400030, China

^*^ hk.xie@ieee.org

**Supplementary Materials:**

Figures S1-S4

Table S1-S4.

References 1-6.


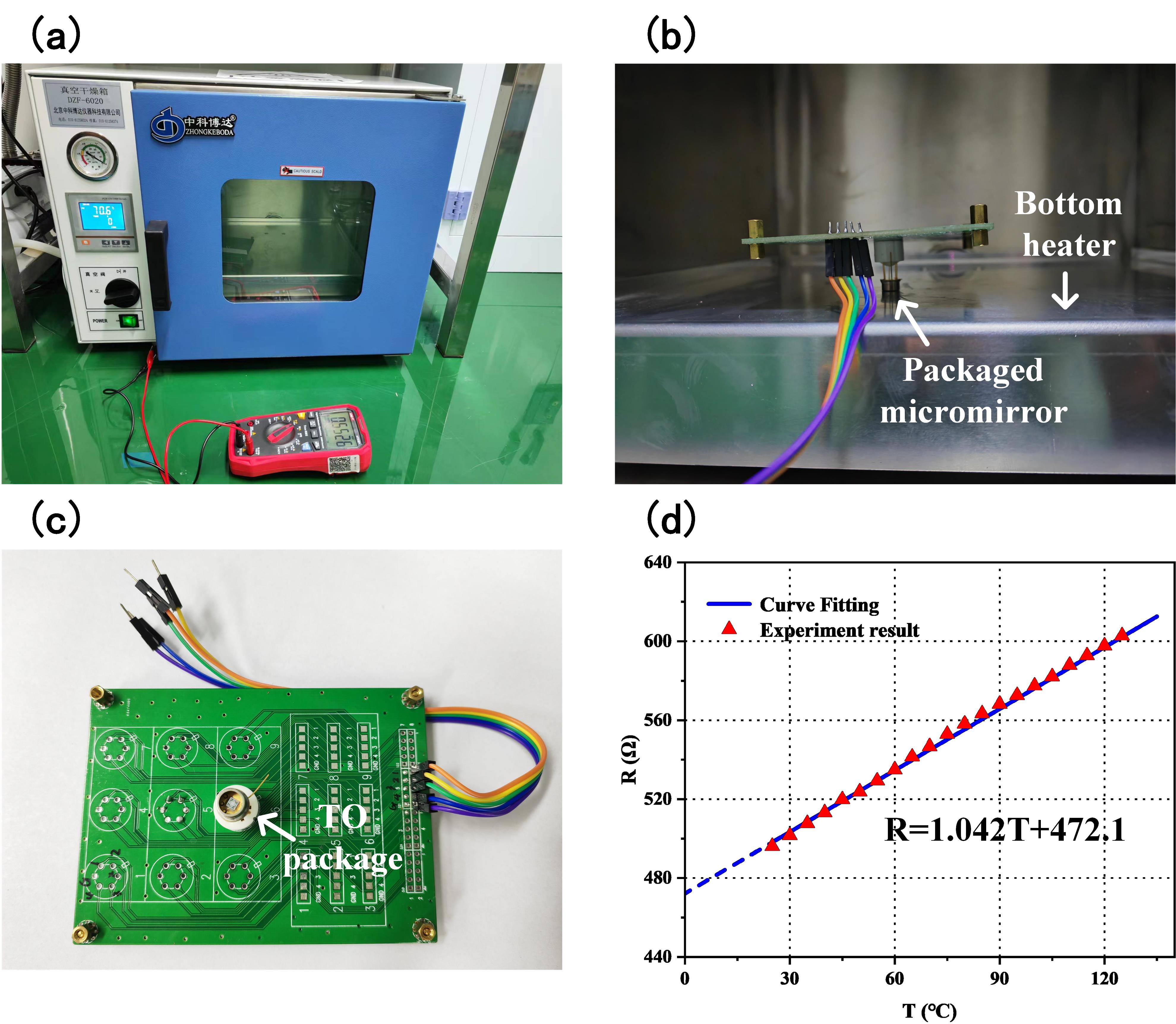


**Figure S1. The test method for temperature coefficient of resistance.** (a) The whole view of the test setup. A temperature-controlled oven is used to set the temperature, and a multimeter is used to measure the resistance value at the specific temperature. (b) A close view of the device in the oven. To maximize the heat concentration on the micromirror, the TO package is inverted on the bottom heating plate. (c) A close view of the PCB circuit board of the micromirror. (d) The relationship between the resistances and the temperature. The measurement error of the resistance is ±1 Ω. The TCR of the resistance can be calculated as the ratio of the slope to the R-axis intercept of its R-T curve^1^, and the value is 0.0022/K.


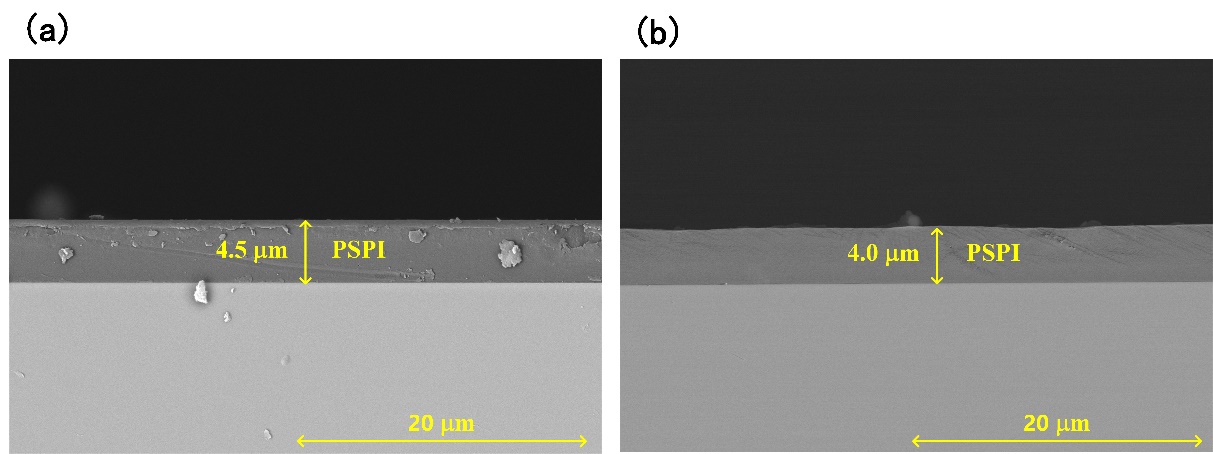


**Figure S2. The thickness of PSPI before and after high-temperature curing.** (a). Before curing. (b). After curing.


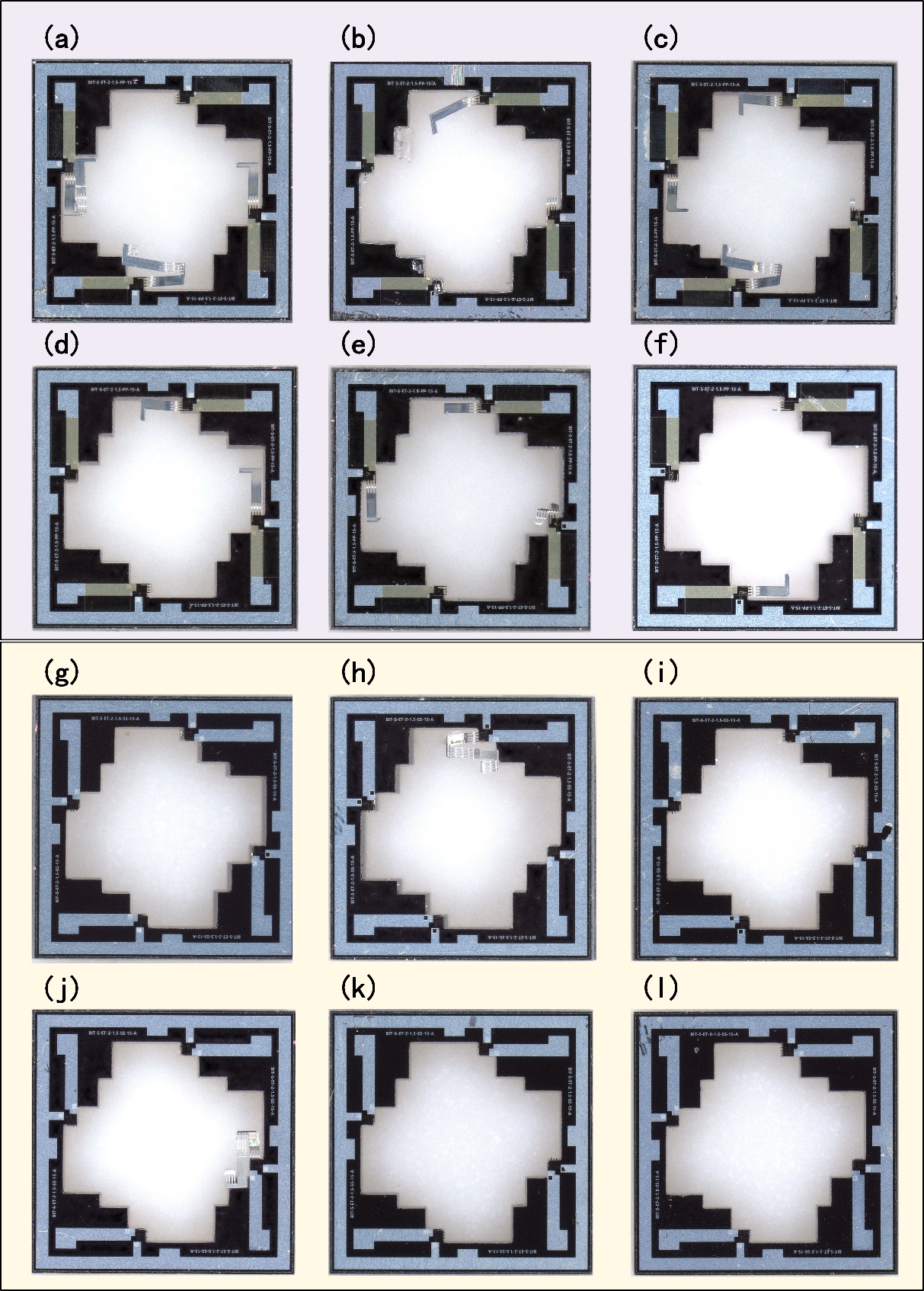


**Figure S3. Damaged pictures of the two types of micromirrors.** (a~f). The PSPI-type micromirrors. (g ~ l). The SiO_2_-type micromirrors.

**The Simulation of the Temperature Distribution on PSPI Beams**

To explore the temperature distribution on the PSPI beam at both ends of a bimorph actuator, a 3D model of one of the actuators has been built in COMSOL software, as shown in Figure S4(a). The structure of the 3D model is consistent with the actual design. The operating voltage is set to 4V DC. The room temperature is set to 20 ℃, and the heat transfer coefficient (h_e_) is set to 1900 W/m^2^K^3^. The detailed temperature distribution on the actuator is shown in Figure S4(b). The temperature on the PSPI at both ends of the actuator shows a gradient distribution, which effectively reduces the heat loss from the actuator to the substrate and the mirror plate. Figures S4(c) and S4(d) show the detailed temperature values of the two ends of the PSPI beam, respectively. When the distance from the monitoring point to the substrate/mirror frame increases, the temperature values increase linearly. The simulation results show that when a 4V DC operating voltage is applied to the actuator. Table S3 provides the thermal properties of the PSPI used in our case. According to the simulation results, when the micromirror is operated at a 4V DC voltage, the maximum temperature in the PSPI beam is about 134 ℃, which is much lower than its 5% thermal weight loss temperature of about 500 ℃^2^. In addition, it is reported that the lifetime of a polyimide film with a thickness of 4.2μm is about 400 hours when the operating temperature is 360 ℃ in the air^4^, which indicates a longer lifetime could be achieved in our case. Further exploration of the PSPI’s thermal failure will be carried out in the future..


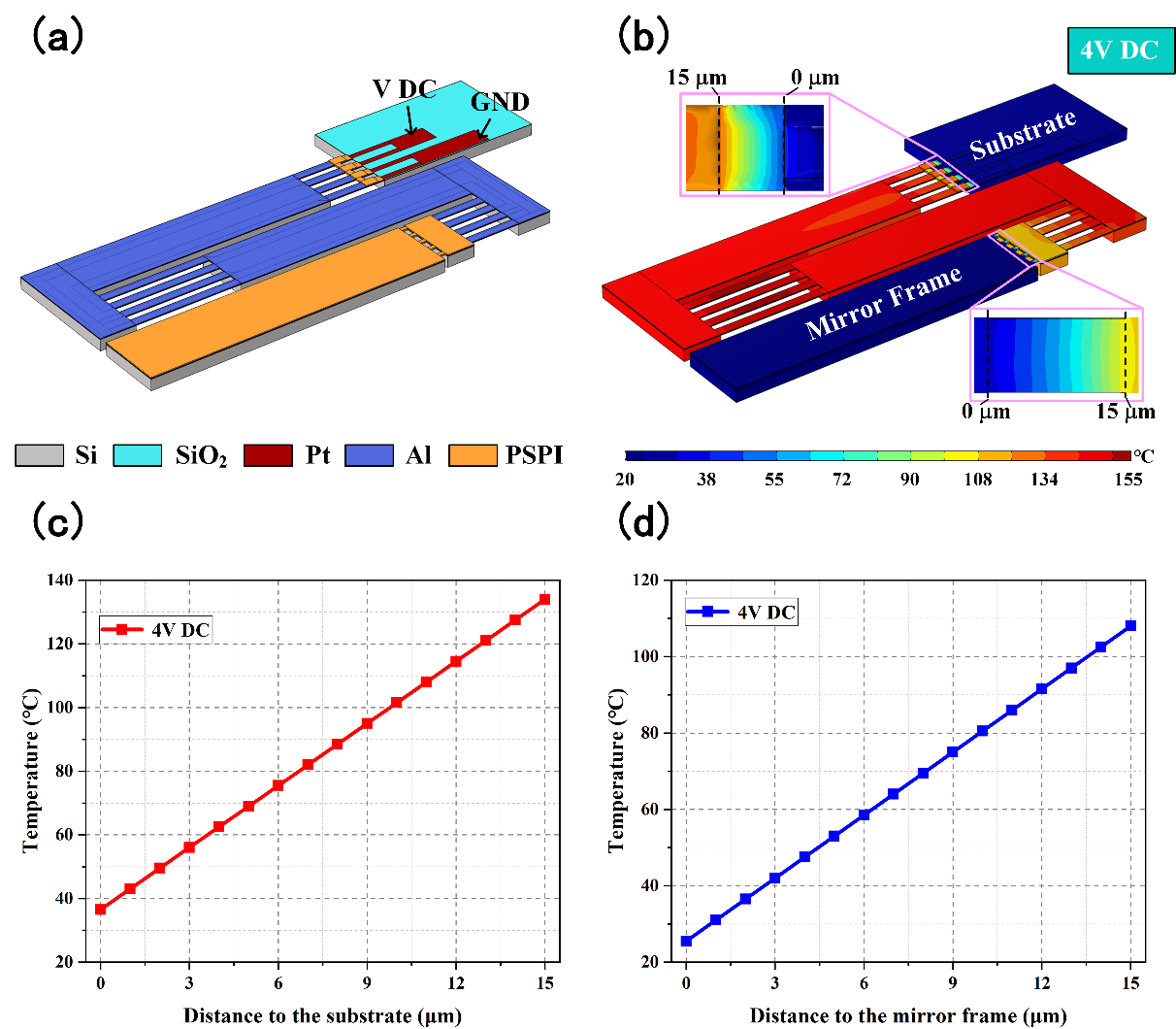


**Figure S4. Simulation results of one actuator of the electrothermal micromirror.** (a) A 3D model of the actuator built in COMSOL software. (b) Temperature distribution of the actuator excited by 4V DC voltage. (c) Temperature value of PSPI beam on the substrate end. (d) Temperature value of PSPI beam on the mirror frame end.

Table S1. Comparison of properties of the selected materials^2,5^

| Material | Young’s modulus (GPa) | CTE (ppm K^-1^) | Thermal conductivity (W m^-1^ K^-1^) | Electrical conductivity (S m^-1^) |
| --- | --- | --- | --- | --- |
| Al | 70 | 23.1 | 237 | 35.5 × 10^6^ |
| SiO_2_ | 70 | 0.5 | 1.4 | Insulator |
| Pt | 168 | 8.8 | 71.6 | 8.9 × 10^6^ |
| PSPI | 2.3 | 20 | 0.15 | Insulator |

Table S2. The simulation results of the micromirrors’ resonance frequency versus the length of the PSPI beam.

| length of the PSPI beam (μm) | resonance frequency (Hz) |
| --- | --- |
| 5 | 213 |
| 15 | 205 |
| 25 | 197 |
| 35 | 192 |
| 45 | 187 |
| 55 | 183 |

Table S3. The resistance and frequency test results of a PSPI-type micromirror in the drop test

| Drop height(cm) | R_1_ (Ω) | R_2_ (Ω) | R_3_ (Ω) | R_4_ (Ω) | f_1_(Hz) | f_2_(Hz) | Microscopic Examination |
| --- | --- | --- | --- | --- | --- | --- | --- |
| 1 | 528.6 | 534.1 | 526.7 | 529.2 | 198 | 388 | OK |
| 3 | 528.7 | 533.4 | 525.7 | 528.3 | 198 | 388 | OK |
| 5 | 529.1 | 535.2 | 528.8 | 530.4 | 198 | 387 | OK |
| 7 | 528.3 | 537.7 | 524.3 | 527.4 | 197 | 386 | OK |
| 9 | 530.1 | 536.3 | 527.1 | 529.7 | 197 | 386 | OK |
| 11 | 531.4 | 533.1 | 529.4 | 531.2 | 195 | 385 | OK |
| 13 | 529.7 | 534.7 | 531.2 | 533.4 | 190 | 383 | OK |
| 15 | 530.4 | 537.4 | 527.4 | 530.6 | 184 | 371 | OK |
| 17 | 533.1 | 536.3 | 534.1 | 532.8 | 177 | 366 | OK |
| 19 | 532.6 | 533.9 | 536.5 | 530.3 | 162 | 344 | OK |
| 21 | 531.8 | 535.2 | 537.1 | 533.4 | 155 | 336 | OK |
| 23 | \ | \ | \ | \ | \ | \ | Fail* |

*The mirror plate detached from the device.

Table S4. Detailed curing temperature parameters of PSPI^2,6^.

| Parameter | Value |
| --- | --- |
| Pre-curing temperature | 120℃, 5min |
| Curing temperature | 150℃, 60min |
|  | 150℃ →180℃, 10min |
|  | 180℃, 60min |
|  | 180℃ →250℃, 20min |
|  | 250℃, 60min |
|  | 250℃ →320℃, 20min |
|  | 320℃, 120min |
| The temperature of 5% weight loss | 500 ℃ |

**Reference**

1. Xiao, L, Ding, Y. Wang, P & Xie, H. Analog-controlled light microshutters based on electrothermal actuation for smart windows. *Opt Express* **28**, 33106 (2020).

2. http://jing-ai.com/a/about/.

3. Zhou, L. et al. Investigation of dynamic thermal behaviors of an electrothermal micromirror. *Sens. Actuators, A*. **263**. 269-275 (2017).

4. Khazaka, J. R., Diaham, S., Locatelli, M. L., Trupin, C. & Schlegel, B. Thermal and thermo-oxidative aging effects on the dielectric properties of thin polyimide films coated on metal substrate. in *2011 Annual Report Conference on Electrical Insulation and Dielectric Phenomena*. 52-55 (IEEE, 2011).

5. Pal, S. & Xie, H. Fabrication of robust electrothermal MEMS devices using aluminum–tungsten bimorphs and polyimide thermal isolation. *J. Micromech. Microeng.* **22**, 115036 (2012).

6. Cai, Z., Ding, Y., Wu, Z., Zhang, Z., Su, Y. & Chen. Z. An all-wet, low cost RDL fabrication process with electroless plated seed/barrier layers. in *2021 IEEE International Interconnect Technology Conference (IITC)*. 1-4 (IEEE, 2021).
